# PD05 Safety Profile Of Miltefosine In The Treatment Of Cutaneous Leishmaniasis: An Analysis Of Use In Minas Gerais, Brazil

**DOI:** 10.1017/S0266462325101438

**Published:** 2025-12-29

**Authors:** Sarah Silva, Laís Ribeiro, Janaína Carvalho, Mell Saliba, Gláucia Cota

## Abstract

**Introduction:**

The incorporation of a new technology in the health system requires extensive planning, actions towards its implementation, and monitoring of its effectiveness and safety in the population. The objective of this study was to evaluate the safety profile of miltefosine in the treatment of cutaneous leishmaniasis during the initial implementation phase.

**Methods:**

Data from all patients who used miltefosine in the state of Minas Gerais between May 2021 and July 2023 were analyzed. Patient data were collected from the reporting and clinical monitoring form. Adverse events were classified according to the Medical Dictionary for Regulatory Activities; severity was categorized according to World Health Organization criteria and intensity according to the Division of AIDS. Descriptive analyses presenting measures of central tendency and dispersion for events were used, as well as multivariate analysis for explanatory variables and outcomes of interest.

**Results:**

The frequency of adverse events was 77.1 percent, 3.8 percent of which were severe, resulting in six patients requiring hospitalization. Gastrointestinal manifestations were the most frequent clinical adverse event, followed by musculoskeletal manifestations. The multivariate analysis indicated an association between diabetes and the occurrence of adverse clinical events. The most common laboratory alteration was elevated serum creatinine, which was significantly associated with arterial pressure, age, and mucosal clinical form. There were no records of pregnancy among the treated women. The rate of early treatment discontinuation was 11.8 percent, which was associated with age and an alteration in baseline serum creatinine level.

**Conclusions:**

This study demonstrated the high frequency of adverse events that occur with miltefosine, predominantly gastrointestinal and renal function alterations, highlighting that pharmacovigilance strategies need to be implemented routinely. These data are essential for managing health technologies and supporting new decisions, thus contributing to patient safety.

